# Meeting report of the RNA Ontology Consortium January 8-9, 2011

**DOI:** 10.4056/sigs.1724282

**Published:** 2011-04-29

**Authors:** Amanda Birmingham, Jose C. Clemente, Narayan Desai, Jack Gilbert, Antonio Gonzalez, Nikos Kyrpides, Folker Meyer, Eric Nawrocki, Peter Sterk, Jesse Stombaugh, Zasha Weinberg, Doug Wendel, Neocles B. Leontis, Craig Zirbel, Rob Knight, Alain Laederach

**Affiliations:** 1 Thermo Fisher Scientific, Lafayette, CO, USA; 2 Department of Chemistry and Biochemistry, University of Colorado, Boulder, CO, USA; 3 Argonne National Laboratory, Argonne, IL, USA; 4 Department of Ecology and Evolution, University of Chicago, Chicago, IL, USA; 5 DOE Joint Genome Institute, Walnut Creek, CA, USA; 6 Computation Institute, University of Chicago, Chicago, IL, USA; 7 Janelia Farm Research Campus, Howard Hughes Medical Institute, Ashburn, VA, USA; 8 Wellcome Trust Sanger Institute, Wellcome Trust Genome Campus, Hinxton, Cambridge, UK; 9 Department of Molecular, Cellular and Developmental, Yale University, New Haven, CT, USA; 10 Howard Hughes Medical Institute, Yale University, New Haven, CT, USA; 11 Department of Chemistry, Bowling Green State University, Bowling Green, OH, USA; 12 Department of Mathematics and Statistics, Bowling Green State University, Bowling Green, OH, USA; 13 Howard Hughes Medical Institute, Boulder, CO, USA; 14 Department of Biology, University of North Carolina at Chapel Hill, Chapel Hill, NC, USA

## Abstract

This report summarizes the proceedings of the structure mapping working group meeting of the RNA Ontology Consortium (ROC), held in Kona, Hawaii on January 8-9, 2011. The ROC hosted this workshop to facilitate collaborations among those researchers formalizing concepts in RNA, those developing RNA-related software, and those performing genome annotation and standardization. The workshop included three software presentations, extended round-table discussions, and the constitution of two new working groups, the first to address the need for better software integration and the second to discuss standardization and benchmarking of existing RNA annotation pipelines. These working groups have subsequently pursued concrete implementation of actions suggested during the discussion. Further information about the ROC and its activities can be found at http://roc.bgsu.edu/.

## Introduction

The RNA Ontology Consortium is an international coalition of RNA researchers working to develop controlled vocabularies pertaining to RNA function and based on RNA primary sequence, secondary structure, and tertiary structure. Launched in 2005 with funding from the National Science Foundation, the ROC includes nine working groups addressing the ontology needs of RNA domains ranging from backbone conformation to multiple sequence alignment. The Consortium has published an RNA Ontology (RNAO) [1] and an RNA Structure Alignment Ontology [2] for use by the wider research community, and where applicable has integrated these resources with other relevant ontologies such as the Sequence Ontology (SO) [3], Chemical Entities of Biological Interest (CheBI) [4], and the Ontology for Biomedical Investigations (OBI) [5].

This workshop furthered the goals of the consortium by making progress on several of the RNA structure mapping concepts elaborated upon during previous ROC meetings in Kona and in Strasbourg. In addition, it brought together members of the ROC with members of the Genomic Standards Consortium (GSC) [6], an open-membership organization working towards 1) implementing new genomic standards, 2) capturing and exchanging information expressed in these standards and 3) harmonizing information collection and analysis efforts across the wider genomics community. The aim of developing links with the GSC was to inform developers and curators of genomic and metagenomic software and resources of the standards for representing RNA secondary and 3D structures and alignments developed by ROC and to encourage collaboration to improve genomic annotations.

The overall structure of the meeting consisted of a working dinner, software presentations, a roundtable discussion on annotation challenges, and assembly of working groups to address these challenges. The major annotation issues that were identified included: 1) How can information that is useful for drawing/representing RNA structures be exchanged among different software programs and incorporated into different genomic and metagenomic web sites for display to end users? 2) How can information about covariance models automatically be used to infer correspondence groups? 3) How can information about structure mapping experiments be captured to facilitate automatic secondary structure inference and propagated and displayed in a way that is useful to end users?

## Software Presentations

*Eric Nawrocki* provided an introduction to structural RNA homology search using Infernal (INFERence of RNA ALignment) [7]. He noted that searching DNA sequence for noncoding RNA (ncRNA) molecules is more difficult than searching for proteins, because ncRNA molecules are generally shorter than protein molecules and their alphabet is smaller, so conserved secondary structure of RNAs provides useful additional signal. Infernal scores the combination of consensus sequence and consensus structure using covariance models; this technique produces results that are more sensitive than either BLAST or basic hidden-Markov model-based approaches. A major drawback of the current implementation of Infernal is that it is computationally intensive, but acceleration remains a major goal of the project.

*Jesse Stombaugh* presented a live demonstration of Boulder ALE (ALignment Editor) [8], a tool for manual examination and adjustment of multiple sequence alignments. Such manual processing is often critical to developing gold-standard alignments that can be used as test cases for automated alignment tools. Among other features, Boulder ALE allows users to examine isostericity of basepairs in the alignment relative to a reference secondary or 3D structure, to show or hide entire sequence features, to rearrange sequences within the alignment, and to shift nucleotides (e.g. by insertion/deletion of gaps in aligned sequences) and view the effect of these additions on alignment quality. The program currently takes inputs in FASTA format, but will soon be modified to take Stockholm format [9] inputs for increased interoperability with other alignment software.

*Zasha Weinberg* introduced R2R, software new application to aid in drawing publication-quality figures for single and consensus RNA structures [10]. The goal of this software is to create structure drawing with both aesthetic layout and embedded annotation while minimizing the requirement for manual work in a graphics program such as Adobe Illustrator. One of the  notable features of R2R is its ability to gracefully depict information on variability of consensus structures, such as hairpins of variable lengths or junctions that join variable numbers of stems. Currently the software is implemented as a command-line tool that takes input in Stockholm format and produces PDF or SVG images, as well as intermediate files defining the annotation and layout features.

## Day One Discussion

After the three software presentations, Day One proceeded with an extensive round-table discussion of the role of the RNAO in annotation pipelines for RNA discovery. While protein (gene) annotation is a relatively mature field, RNA annotation is much less mature. Given this fact, there is an opportunity to implement ontological structure and support from the very beginning of the standardization of the field. The discussion focused on identifying ontological needs for end users who create RNA annotations or integrate them into larger genome resources.

In such a scenario, one key use for ontologies would be to identify the provenance of RNA sequences incorporated into annotation pipelines and describe the techniques used to generate these sequences. It was noted that researchers performing RNA annotation on sequences generated for protein-level work (i.e. genome and metagenome sequences) may be unaware of quality issues associated with these sequences. For example, some sequencing facilities pre-filter genomic and metagenomic sequences to remove low-quality sequences before distributing the sequences to researchers. However, the researchers may not be given the parameters and software tools that were used for the pre-filtering step and be unaware that filtering steps had been applied with a potential loss of RNA sequences as a consequence.

This caveat also applies to RNA structure information. It was agreed that evidence codes, analogous to those applied to gene annotations, to describe the process by which publicly available RNA secondary and 3D structures were constructed would be extremely useful. In the ideal situation, service(s) would exist that could be queried with a structure identifier and would return both an evidence code summarizing the quality of and backing for the structure model as well as a persistent identifier linking to more detailed metadata. Participants also argued that improving interoperability and comparability of annotation tools and pipelines is a major need. Day Two was therefore structured to create time for two new working groups to form and meet, the first to address the need for better software integration and the second to discuss standardization and benchmarking of existing RNA annotation pipelines.

## Day Two Discussion

### Software Integration Group

This working group includes the developers of various tools for building, visualizing and manipulating RNA alignments and structure mapping information (Infernal, R2R, BoulderALE), as well as the developers of the Single Nucleotide Resolution Nucleic Acid Structure Mapping (SNRNASM) standard for capturing information, describing and reporting single-nucleotide resolution nucleic acid structure mapping assays [11,12]. The main objectives of the working group were to adapt the Stockholm format to provide information about visualization for end users and to automate the production of annotations of correspondences in alignments, secondary and tertiary structure annotations, and molecular functions. One key idea was that information about visualization is itself an annotation. It was agreed that, WYSIWIG support might be added in the future, for now it would be easier to provide presentation information through a set of linked tools using a common file format. RNAO annotations will be referenced according to their unique identifiers in the RNAO and, where appropriate, with citations to papers describing them.

One issue that was identified was that some databases such as Rfam [13] do not provide a way to store presentation information. This issue is solved by the extension of the Stockholm format, as Rfam already provides files in this format for download. Because modifying Stockholm files could conflict with data from the original authors, it was agreed that only presentation hints would be added, rather than changing  the original data. However, it was agreed that using BoulderALE to curate a set of alignments, build better Infernal models, and to draw pictures with R2R would be a useful research project. Collaborative visits are planned between the authors of the BoulderALE and the R2R programs to iron out these specific issues.

As a result of the meeting, the authors of the three packages (Infernal, R2R and BoulderALE) agreed to modify their programs to use the extended format. It was also decided to encourage the developers of other software, notably VARNA [14], to adopt the extended format BoulderALE will be extended to support selection of a subset of sequences and dispatch them to the Infernal cmbuild to create a custom covariance model, to re-align a set of sequences with Infernal, and to read and display alignment confidence scores from Infernal. Additionally, BoulderALE will be extended to provide the capability to annotate R2R features on the alignment and export these features to R2R. A set of extensions to the Stockholm format was identified and specific tasks allocated to each participant.

### Annotation Standardization Group

Consistent RNAannotation is a major need of both producers of genomic and metagenomic data, and researchers analyzing vast numbers of sequences currently being generated. It was proposed that standardization of such annotations could be brought under the umbrella of the GSC.

A clear path forward to standardization of RNA annotation would be to set up an open competition in which existing annotation pipelines perform annotation of RNAs on a gold-standard data set. The pipeline with the best results, i.e. the one that correctly identifies and annotates most known RNAs, would be the ‘gold standard’ for that year, and the results of the competition would be made available to all pipeline developers for use in improving their tools. One point raised was that most current pipelines include significant pre-filtering steps, which are frequently not made public with the pipeline or described in detail. Such a competition could address the question of whether or not these pre-filtering differences meaningfully affect results and, if so, encourage open discussion of these steps.

The working group included researchers with access to completed, but currently unpublished genome data that could serve as the gold-standard data set. In addition, members began investigating potential funding opportunities to support the administration, implementation, and publication of the proposed competition.

## Conclusions

This meeting provided a forum for knowledge exchange and discussion and led to the establishment of two working groups to promote work on software integration and standardization of RNA descriptions , ideally under the auspices of the GSC. We established an extension of the Stockholm format for annotating alignments that is compatible with Infernal, Boulder Ale and R2R. This format, based on that of R2R, will allow people to make 2D diagrams from their alignments, and will be used to improve covariance models in Infernal. Overall, the prospects for improved RNA annotation are bright, and the meeting was extremely productive in bringing together members of previously non-overlapping communities, this time also including several leading members of the GSC. The participants thank the ROC and the NSF for the opportunity to hold this meeting, which all found extremely valuable.
